# Antibacterial interactions, anti-inflammatory and cytotoxic effects of four medicinal plant species

**DOI:** 10.1186/s12906-018-2264-z

**Published:** 2018-07-03

**Authors:** Refilwe G. Kudumela, Lyndy J. McGaw, Peter Masoko

**Affiliations:** 1 Department of Biochemistry, Microbiology and Biotechnology, Faculty of Science and Agriculture, University of Limpopo, Turfloop Campus, Private Bag X1106, Sovenga, Limpopo 0727 South Africa; 2 Department of Paraclinical Sciences, Phytomedicine Programme, Faculty of Veterinary Science, University of Pretoria, Private Bag X04, Onderstepoort, 0110 South Africa

## Abstract

**Background:**

The constant emergence of antibiotic resistant species and the adverse side effects of synthetic drugs are threatening the efficacy of the drugs that are currently in use. This study was aimed at investigating the possible antibacterial interactions, anti-inflammatory and cytotoxic effects of selected medicinal plants based on their traditional usage.

**Methods:**

The acetone extracts of four plant species were assessed independently and in combination for antibacterial activity using microdilution assay and the sum of the fractional inhibitory concentration (FIC) was calculated. The ability of *Dombeya rotundifolia* and *Schkuhria pinnata* extracts to inhibit the production of reactive oxygen species (ROS) in LPS induced RAW 264.7 macrophage cells was evaluated using Dichloro-dihydro-fluorescein diacetate (H_2_DCF-DA) assay to determine anti-inflammatory potential and the toxicity on African green monkey kidney (Vero) cells was evaluated using 3-(4,5-dimethylthiazol-2-yl)-2,5-diphenyl tetrazolium bromide (MTT) assay.

**Results:**

The antibacterial efficacies of the different combinations of *Schkuhria pinnata* (A), *Commelina africana* (B), *Dombeya rotundifolia* (C) and *Elephantorrhiza elephantina* (D) plants varied from combination to combination. Synergistic effects were only exhibited against *P. aeruginosa*, while the antagonistic effects were only observed against *E. coli*. Both *S. pinnata* and *D. rotundifolia* demonstrated anti-inflammatory potential by inhibiting the production of ROS in a dose dependant manner. The cytotoxicity of the plants (LC_50_ values) ranged from < 25.0 to 466.1 μg/mL. *S pinnata* extract was the most toxic with the lowest LC_50_ value of < 25.0 μg/mL.

**Conclusions:**

The synergistic interaction observed indicates that combinational therapy may improve biological activity. This report highlights the anti-inflammatory potential of *S. pinnata* and *D. rotundifolia*; which could be exploited in the search for anti-inflammatory agents. However, the cytotoxicity of *S. pinnata* highlights the importance of using this plant with caution.

## Background

The extensive use and over reliance on antibiotics has caused the bacteria to develop resistant genes against the available antibiotics [1]. Some antibiotics have been associated with undesirable side effects, such as; nausea, depression of bone marrow, thrombocytopenic and agranulocytosis [2], therefore medicinal plants are researched as possible new sources of antimicrobial agents with possibly novel mechanisms of action and fewer side effects since they have therapeutic relevance in folklore [3–7]. These medicinal plants are used in the form of herbal remedies which are prepared from one plant or a combination of different plant species [8]. Herbal mixtures containing a combination of different plant species have been reported to have better biological activities than isolated active compounds and herbal mixtures prepared from one plant species [8–10]. Therefore, these could be used to overcome the challenge of antimicrobial resistance [11].

The use of traditional medicine systems to treat various ailments has been in existence for years and continues to provide the human population with new medicines [12]. A single plant can be used for treatment of more than one type of disease and as a result have multiple medicinal properties. This justifies why it is important to screen for more than one biological activity when screening plants for biological activity. This approach explores and provides information on the overall medicinal properties of specific medicinal plants [11]. The current steroidal and non-steroidal anti-inflammatory drugs present adverse side effects, thus, exploring plants as an alternative has been increasing significantly [13].

Medicinal plants are assumed to be safe based on their long history of use in the treatment and management of diseases [14]. However, the use of these plants may be associated with irritation of the gastrointestinal tract, destruction of red blood cells, and damage of the heart and kidney [15]. Therefore, this necessitates the need for cytotoxicity evaluation of medicinal plants used in ethnopharmacology. The present study investigated the antibacterial interactions, anti-inflammatory and cytotoxic effects of the four selected plants. This was motivated by the results obtained in our previous work on phytochemical screening, antioxidant and antibacterial effects of the same plants [16]. The plants selected for this study include; *Commelina africana* L. var. *africana* (Commelinaceae) which is used traditionally for the treatment of venereal diseases [17]; *Dombeya rotundifolia* (Hochst.) Planch*.* var. (Sterculiaceae) used traditionally to treat diarrhoea [18]; *Elephantorrhiza elephantina* (Burch.) Skeels (Leguminosae) used for treatment of pneumonia and tick-borne diseases in cows [19] and *Schkuhria pinnata* (Lam.) Kuntze ex Thell (Asteraceae) which is used for treatment of stomach ache [20].

## Methods

### Chemicals

Acetone (Sigma Aldrich, SA), Ampicillin (Sigma Aldrich, SA), nutrient broth (Oxiod), Curcumin (Adcock-Ingram), Dichloro-dihydro-fluorescein diacetate (H_2_DCF-DA) (ThermoFisher Scientific), Lipopolysaccharide (LPS) (ThermoFisher Scientific), RPMI-1640 medium (Whitehead Scientific), Phosphate Buffered Saline (PBS) (White Scientific), foetal bovine serum (FBS) (Hyclone, Thermo Scientific), Minimum Essential Medium (MEM, Whitehead Scientific) gentamicin (Virbac) 3-(4, 5-dimethylthiazol-2-yl)-2, 5-diphenyltetrazolium bromide (MTT) (Sigma Aldrich, SA), foetal calf serum (Highveld Biological).

### Plant collection and preparations

The leaves of (*Commelina africana* L. var. *africana* (UNIN 12295)*, Dombeya rotundifolia* (Hochst.) Planch*.* var. *rotundifolia* (UNIN 12296)*, Elephantorrhiza elephantina* (Burch.) Skeels (UNIN 12297) and the whole plant excluding the roots of *Schkuhria pinnata* (Lam.) Kuntze ex Thell (UNIN 12298) were collected in April 2015 at University of Limpopo, South Africa. The specimens were deposited at the Larry Leach Herbarium (UNIN) for authentication. Plant collection was based on ethnopharmacological information provided by traditional healers in Limpopo Province. The plants were dried at room temperature, protected from light and later ground to fine powder using a blender (Waring Laboratory Blender LB20ES). The plant materials (10 g) were extracted using acetone (100 mL) (Sigma-Aldrich, S.A) and the supernatants of each plant material were filtered through Whatman No.1 filter paper into pre-weighed vials and the filtrates were dried under a stream of air. The dried filtrates yielded 0.3 g, 0.7 g, 1.08 g and 0.5 g for *C. africana*, *D. rotundifolia*, *E. elephantina* and *S. pinnata* respectively.

### Microorganisms used in this study

Two Gram-positive bacteria (*Staphylococcus aureus* ATCC 29213 and *Enterococcus faecalis* ATCC 29212) and 2 Gram-negative bacteria (*Escherichia coli* ATCC 28922 and *Pseudomonas aeruginosa* ATCC 27853) were used. These are major causes of nosocomial infections in hospitals [21] and are mainly the strains recommended for use by the National Committee for Clinical Laboratory Standards [22]. The bacterial species were maintained on nutrient agar at 4 °C. The cells were inoculated and incubated at 37 °C in nutrient broth for 12 h prior to screening tests.

### Antibacterial interaction activity

The antibacterial activity interactions of the four selected plants were investigated by the determination of the minimum inhibitory concentration (MIC) of each plant independently and in combination using the micro-broth dilution assay described by Eloff [23]. Stock solutions (10 mg/mL) of acetone extracts of each plant were prepared by re-dissolving the extracts in acetone. For 1:1 test combinations, 50 μL of each of the two extracts were mixed to make up a volume of 100 μL in the first wells of a 96-well microtitre plate. Each extract contributed 33.3 μL and 25 μL for the 1:1:1 and 1:1:1:1 combinations respectively, to make up 100 μL in the first wells of a 96-well microtitre plate [8]. Two-fold serial dilutions of these extracts and ampicillin (positive control) (2.5 mg/mL to 0.02 mg/mL) were prepared in 96-well microtitre plates. The effects of the extracts were tested against 100 μL of each pathogen at the following densities of bacterial cultures: *S. aureus*, 2.6 X 10^12^ colony-forming units (CFU/mL); *E. faecalis*, 1.5 X 10^10^ CFU/mL; *P. aeruginosa*, 5.2 X 10^13^ CFU/mL; *E. coli*, 3.0 X 10^11^ CFU/mL. The microtitre plates were incubated at 37 °C overnight. Thereafter, 40 μL of 0.2 mg/mL iodonitrotetrazolium chloride (Sigma-Aldrich) was added to each well and the plates were re-incubated for a further 30 min at 37 °C for *S. aureus* and *P. aeruginosa*, 1.5 h for *E. coli*, and 24 h for *E. faecalis*. The formation of a red-pink color signified microbial growth. All samples were assayed in triplicates and acetone was used as a negative control. The synergistic or antagonistic interactions between the plants were investigated by determining the Fractional inhibitory concentrations (FIC). These were calculated for 1:1 combinations of the plants with the equation below, where (i) and (ii) represented the different 1:1 plant combinations [24]. The FIC index was expressed as the sum of FIC _(i)_ and FIC _(ii)_ and this was used to classify the interaction as either synergistic (≤0.50), additive (0.50–1.00), indifferent (> 1.00–4.00) or antagonistic (> 4.00) [25].

$$ \mathrm{FIC}\left(\mathrm{i}\right)\frac{\mathrm{MIC}\ \mathrm{of}\ \left(\mathrm{a}\right)\mathrm{in}\ \mathrm{combination}\ \mathrm{with}\ \left(\mathrm{b}\right)}{\mathrm{MIC}\ \mathrm{of}\ \left(\mathrm{a}\right)\mathrm{in}\mathrm{dependently}} $$

$$ \mathrm{FIC}\left(\mathrm{ii}\right)\frac{\mathrm{MIC}\ \mathrm{of}\ \left(\mathrm{b}\right)\mathrm{in}\ \mathrm{combination}\ \mathrm{with}\ \left(\mathrm{a}\right)}{\mathrm{MIC}\ \mathrm{of}\ \left(\mathrm{b}\right)\mathrm{in}\mathrm{dependently}} $$

### Anti-inflammatory activity assay using Dichloro-dihydro-fluorescein diacetate H_2_DCF-DA assay

Based on the reported high antioxidant and antibacterial activities of *D. rotundifolia* and *S. pinnata* respectively [16] the anti-inflammatory activity assay of the two plants were investigated using the H_2_DCF-DA assay as described by Sekhar et al.*,* [26] with slight modifications. This assay uses stimulants such as lipopolysaccharide (LPS) to induce oxidative stress and Dichloro-dihydro-fluorescein diacetate (H_2_DCF-DA) to detect the presence of reactive oxygen species.. In the presence of reactive oxygen species H_2_DCF-DA is oxidized to fluorescent 2, 7-dichlorofluorescein (DCF). Two hundred microliters of cells (RAW 264.7 macrophages) obtained from the American Type Culture Collection (ATCC) in RPMI-1640 was seeded in a 96-well plate. The cells were incubated at 37 °C, 5% CO_2_ overnight to attain confluency. The medium was removed and the cells were washed with Phosphate Buffered Saline (PBS). Cells were exposed to 100 μL of acetone extracts (8 mg/mL, 0.64 mg/mL, and 0.32 mg/mL) of *D. rotundifolia* and *S. pinnata* and 20 μL of LPS for 24 h. Following incubation, the medium was aspirated and fresh medium without Foetal Bovine Serum (FBS) was added and the cells were stained with 100 μL of 20 μM of H_2_DCF-DA and incubated for 30 min in the dark. The fluorescence was measured at an excitation wavelength of 480 nm. Curcumin (50 μM) and untreated cells were used as positive and negative controls respectively.

### Cytotoxicity assay

The toxic effects of the selected plants on African green monkey kidney (Vero) cells obtained from the culture collection of the Department of Veterinary Tropical Diseases (University of Pretoria) was determined by the 3-(4, 5-dimethylthiazol-2-yl)-2, 5-diphenyltetrazolium bromide (MTT) assay [27]. The cells were maintained in Minimum Essential Medium (MEM, Whitehead Scientific) supplemented with 0.1% gentamicin (Virbac) and 5% foetal calf serum (Highveld Biological). The cell suspension (5 × 10^4^ cells/mL) was seeded in a sterile 96-well microtitre plate and incubated for 24 h at 37 °C in 5% CO_2_ for the cells to attach. The MEM was aspirated and the cells were washed with 150 μL phosphate buffered saline (PBS, Whitehead Scientific). The cells were treated with different concentrations of the extracts (1–0.025 mg/mL) prepared in MEM. The microtitre plates were incubated for 48 h with the extracts in the same conditions as described earlier. Untreated cells were included as a negative control. After treatment, the treatment medium was aspired and replaced with 200 μL of fresh MEM and then 30 μL of MTT (5 mg/mL) in PBS (Sigma) and the plates were incubated further for 4 h at 37 °C. The medium was removed and replaced with 50 μL of DMSO to dissolve the MTT formazan crystals. The absorbance was measured in a microplate reader (BioTek Synergy) at 570 nm. Cytotoxicity was expressed as the concentration of test sample resulting in a 50% reduction of absorbance compared to untreated cells (LC_50_ values). All the analysis was made in quadruplicate. The selectivity index (SI) was expressed as LC_50_/ MIC value.

### Statistical analysis

Each experiment was performed in triplicates and the results were expressed as mean values. Linear regression analysis was used to calculate LC_50_ values. Microsoft Excel® was used to enter and capture data. Various graphs and tables were extracted from this data. Data was then exported to SPSS for further analysis. The MIC for each microorganism was analyzed using one-way analysis of variance (ANOVA). *P* value < 0.05 was considered as significant. SPSS 25.0 was employed for statistical analysis.

## Results

### Antibacterial interaction activity assay

When the plants were combined in a 1:1:1 combination potent activities were observed when *S. pinnata* was combined with *C. africana* and *D. rotundifolia* (combination A + B + C) against *E. coli* (0.09 ± 0.04 mg/mL) and *P. aeruginosa* (0.06 ± 0.02 mg/mL). Meanwhile, the combination with *D. rotundifolia*, *S. pinnata*, and *E. elephantina* (C + A + D) exhibited potent activities against *P. aeruginosa* (0.07 ± 0.04 mg/mL) only (Table 1). When all the selected plants were combined (combination A + B + C + D) the efficacy against all the tested bacteria except for *E. coli* was enhanced with average MIC values lower than the MIC values of the plants independently. Ampicillin was used as positive control and its MIC values ranged from 0.02 to 0.08 mg/mL. Using ANOVA test (one way ANOVA), the mean difference between the MIC values of some of the acetone extracts combination (A + B; A + B + C; A + B + C + D and C + A against all tested pathogens was statistically significant (*p* < 0.05).

**Table 1:** Antibacterial interaction effects with standard deviation of the acetone extracts of the different combinations of the selected plants against selected bacterial species

Combinations	Minimum Inhibitory Concentration (mg/ml)			
	*Escherichia coli*	*Pseudomonas aeruginosa*	*Enterococcus faecalis*	*Staphylococcus aureus*
A	0.84 ± 0.21	0.27 ± 0.05	1.67 ± 0.83	1.67 ± 0.83
A + B	0.16 ± 0.00	0.04 ± 0.00	0.63 ± 0.00	0.53 ± 0.10
A + B + C	0.09 ± 0.04	0.06 ± 0.02	0.63 ± 0.00	0.32 ± 0.00
A + B + C + D	0.13 ± 0.03	0.07 ± 0.04	0.63 ± 0.00	0.27 ± 0.53
B	0.03 ± 0.01	0.53 ± 0.10	1.67 ± 0.83	0.84 ± 0.00
B + C	0.13 ± 0.03	0.67 ± 0.59	0.63 ± 0.00	0.32 ± 0.00
B + C + D	0.13 ± 0.03	0.76 ± 0.59	0.63 ± 0.00	0.21 ± 0,05
C	0.52 ± 0.36	0.42 ± 0.10	1.25 ± 0.00	0.63 ± 0.00
C + A	0.73 ± 0.27	0.04 ± 0.00	1.25 ± 0.00	0.84 ± 0.21
C + A + D	0.52 ± 0.36	0.07 ± 0.04	1.04 ± 0.21	0.84 ± 0.21
D	1.04 ± 0.21	0.42 ± 0.10	1.67 ± 0.83	0.84 ± 0.21
D + C	0.63 ± 0.00	0.04 ± 0.00	1.67 ± 0.83	0.84 ± 0.21
D + A + B	0.16 ± 0.00	0.67 ± 0.59	0.63 ± 0.00	0.27 ± 0.5
Ampicillin	0.03	0.02	0.03	0.08

Keywords

A = *Schkuhria pinnata*B = Commelina africanaC = Dombeya rotundifoliaD = Elephantorrhiza elephantina

The Fractional Inhibitory Concentration (FIC) values were calculated as outlined above for the 1:1 combinations to establish any synergistic or antagonistic interactions. Synergistic effects were only exhibited against *P. aeruginosa* with 0.22, 0.24, and 0.19 FIC index values for the A + B, C + A and C + D combinations, respectively. Meanwhile, the antagonistic effects were only observed against *E. coli* with 5.52 and 4.58 FIC index values for combinations A + B and B + C respectively (Table 2).

**Table 2:** Fractional inhibitory concentration indexes of the 1:1 combinations of the selected plants

Microorganisms	A + B	B + C	C + A	C + D
*Escherichia coli*	5.52	4.58	2.27	1.82
*Pseudomonas aeruginosa*	0.22	2.86	0.24	0.19
*Enterococcus faecalis*	0.75	0.88	1.75	2.34
*Staphylococcus aureus*	0.95	0.89	1.84	2.33

Keywords

A = *Schkuhria pinnata*B = Commelina africanaC = Dombeya rotundifoliaD = Elephantorrhiza elephantina

### Anti-inflammatory activity

**Fig. 1:**
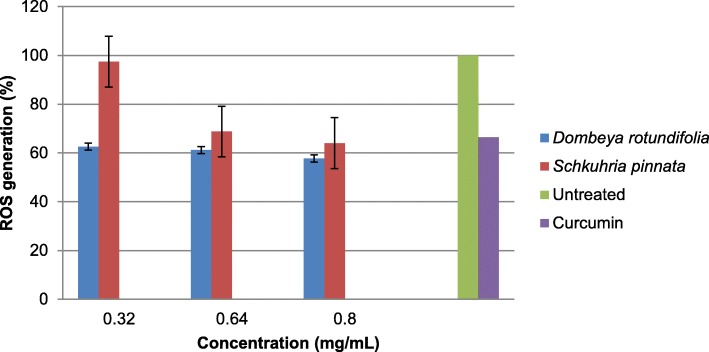
The effect of two of the selected plants on ROS generation inhibition activity in LPS induced RAW 264.7 macrophage cells

The plant extracts inhibited ROS generation in a dose dependant manner. The inhibition was higher in *D. rotundifolia* than in *S. pinnata* (Fig. 1)*.* Curcumin (50 μM) was used as a positive control and all the plants had better anti-inflammatory potential than curcumin at the highest concentration tested. Potent activities were observed in *D. rotundifolia* even at the lowest concentration tested.

### Cytotoxicity assay

The cytotoxicity of the acetone extracts of the selected plants ranged from < 25 to 466.1 μg/mL. The American National Cancer Institute (NCI) described an LC_50_ < 30 μg/mL for plant extracts as a cut off point for cytotoxicity after 72 h of exposure [28]. Therefore, *S. pinnata* extract was highly toxic to the Vero cells with the lowest LC_50_ (< 25 μg/mL) that is outside the cut-off point (Table 3).

**Table 3:** Cytotoxicity, Minimum Inhibitory Concentrations (MIC), and selectivity index (SI) of the acetone extracts of the selected plants

Plant species	LC_50_ (μg/mL)	MIC values (μg/mL)				Selectivity index (SI)			
		*E. coli*	*P. aeruginosa*	*E. faecalis*	*S. aureus*	*E. coli*	*P. aeruginosa*	*E. faecalis*	*S. aureus*
*Schkuhria pinnata*	< 25.0	320	640	1250	1250	0.08	0.04	0.02	0.02
*Dombeya rotundifolia*	466.1	320	1250	1250	1250	1.46	0.37	0.37	0.37
*Elephantorrhiza elephantina*	416.4	640	2500	2500	2500	0.17	0.17	0.17	0.17
*Commelina africana*	441.1	20	2500	2500	2500	22.06	0.18	0.18	0.18

## Discussion

Most traditional healers in South Africa often combine different plants in herbal mixtures. This method has been proposed to be a better way of approaching antimicrobial resistance problem [11]. Plants used in this study, have been reported to have antibacterial activities independently against the tested bacteria [16], hence we here evaluated the possible antibacterial interactions between the plants to see if such combination will potential their antimicrobial activity. To achieve this, the Minimum Inhibitory Concentration (MIC) values of acetone extracts of the selected plants individually and in combination were determined (Table 1). As suggested by Ríos and Recio [29] this study highlights only the MIC values of less or equal to 0.1 mg/mL as these are said to be noteworthy.

Overall the Gram-negative bacteria were more sensitive to the combinations than the Gram-positive bacteria. The difference in sensitivity for these bacteria may be attributed to the difference in membrane morphology [30]. This suggests that the problem of antimicrobial resistance of the Gram-negative strains may be addressed with combinational therapy of these plants. In most cases it is assumed that when two plants are combined synergism is likely to occur [31]. The plants contained in the combinations in this study are used for treatment of various infections and they have been screened for antimicrobial properties individually [16, 32–35]. Mpofu [31] also reported on the synergistic interactions of the 1:1 combinations of *Elephantorrhiza elephantina* and *Pentanisia prunelloides* aqueous extracts against *E. coli* and *E. faecalis*. As such, one could advice on the combination of these plants since combination of plant extracts often offer a wide range of biological activities [36].

There are substantial numbers of reports linking ROS production to inflammation and related diseases [26, 37]. As such, the effects of *S. pinnata* and *D. rotundifolia* acetone extracts on the inhibition of ROS production were investigated in LPS induced RAW 264.7 macrophage cells. This plant was reported to have high free radical scavenging and ferric reducing antioxidant properties [16]. Therefore, the observed anti-inflammatory effect may be attributed to these antioxidant properties. Nevertheless, *S. pinnata* also exhibited ROS inhibition activity at higher concentrations. Therefore, the anti-inflammatory efficacy should be evaluated in vivo before the plant is recommended for any use. Reid et al. [33] also reported on the high inflammatory activity of the ethanol and dichloromethane leaf and bark extracts of *D. rotundifolia*. Meanwhile, Luseba et al. [38] reported that DCM extracts of *S. pinnata* had high inhibitory activity against cyclooxgenase-1 enzyme (COX-1). More often farmers use the same plants to treat different degrees of inflammation and stages of infections [38]. This statement was supported by the potent antibacterial and anti-inflammatory activities of both *S. pinnata* and *D. rotundifolia*.

Many of the plants used in ethnopharmacology to treat various ailments are used with no knowledge of their toxic effect [15]. As such, the toxic effects of the selected plants were evaluated on African green monkey (Vero) cells using MTT assay. This assay is based on the conversion of MTT to an insoluble purple formazan by the mitochondrial succinate dehydrogenase of viable cells [39]. The concentrations of the extracts which resulted in 50% reduction of absorbance compared to untreated cells (LC_50_) are presented in Table 3. Deutschländer et al. [40] also reported the toxicity of the acetone and ethanol extracts of *S. pinnata* on 3 T3-L1 preadipocytes and Chang liver cells. However, McGaw et al. [41] reported that plants containing toxic compounds may have useful biological activities, since toxicity at low doses can be associated with pharmacological activity. Nevertheless, the rest of the plants were less toxic with high LC_50_ values.

Selectivity index was used to relate cytotoxicity and antibacterial activities of plant extracts. These values ranged from 0.02 to 22.06 (Table 3). The plant extracts had low selectivity with an exception of *C. africana* (22.06). The efficacy of biological activity is considered not to be due to toxicity if the selectivity index is ≥10 [42]. Therefore the observed antibacterial activity of acetone leaf extracts of *C. africana* was not due to toxicity. Meanwhile, those of the other plants were probably due to toxicity because of their low selectivity index values. It should be noted that cytotoxicity observed in vitro is not always encountered in vivo. This is probably because when in the biological system, some toxic compounds have the ability to undergo metabolic transformations which leads to the formation of less toxic end products [43].

## Conclusions

This study demonstrated that combinational therapy may be used to address antimicrobial resistance of Gram-negative strains. Furthermore, this report highlights the anti-inflammatory potential of *S. pinnata* and *D. rotundifolia* acetone extracts which could be exploited in the search for plant-based anti-inflammatory agents. However, the cytotoxicity of *S. pinnata* highlights the importance of using this plant with caution.

## Abbreviations

COX: cyclooxygenases
DCFHD-A: dichlorofluorescein diacetate assay
FIC: fractional inhibitory concentration
iNOS: inducible nitric oxide synthase
MEM: Minimum Essential Medium
RNS: Reactive nitrogen species
ROS: Reactive oxygen species
